# The Viable But Non-Culturable State of *Listeria monocytogenes* in the One-Health *Continuum*

**DOI:** 10.3389/fcimb.2022.849915

**Published:** 2022-03-18

**Authors:** Aurélie Lotoux, Eliane Milohanic, Hélène Bierne

**Affiliations:** Université Paris-Saclay, INRAE, AgroParisTech, Micalis Institute, EPIMIC Lab, Jouy-en-Josas, France

**Keywords:** zoonosis, VBNC, dormancy, asymptomatic infections, foodborne pathogen, risk assessment, pregnancy, infectious diseases

## Abstract

Many bacterial species, including several pathogens, can enter a so-called “viable but non-culturable” (VBNC) state when subjected to stress. Bacteria in the VBNC state are metabolically active but have lost their ability to grow on standard culture media, which compromises their detection by conventional techniques based on bacterial division. Under certain conditions, VBNC bacteria can regain their growth capacity and, for pathogens, their virulence potential, through a process called resuscitation. Here, we review the current state of knowledge of the VBNC state of *Listeria monocytogenes* (*Lm*), a Gram-positive pathogenic bacterium responsible for listeriosis, one of the most dangerous foodborne zoonosis. After a brief summary of characteristics of VBNC bacteria, we highlight work on VBNC *Lm* in the environment and in agricultural and food industry settings, with particular emphasis on the impact of antimicrobial treatments. We subsequently discuss recent data suggesting that *Lm* can enter the VBNC state in the host, raising the possibility that VBNC forms contribute to the asymptomatic carriage of this pathogen in wildlife, livestock and even humans. We also consider the resuscitation and virulence potential of VBNC *Lm* and the danger posed by these bacteria to at-risk individuals, particularly pregnant women. Overall, we put forth the hypothesis that VBNC forms contribute to adaptation, persistence, and transmission of *Lm* between different ecological niches in the One-Health *continuum*, and suggest that screening for healthy carriers, using alternative techniques to culture-based enrichment methods, should better prevent listeriosis risks.

## Introduction

*Listeria monocytogenes* (*Lm)* is a ubiquitous Gram-positive bacterium that contaminates food production lines and is pathogenic for humans and various animal species (Bagatella et al., 2021; Quereda et al., 2021). This facultative intracellular pathogen has evolved sophisticated mechanisms to enter, grow and survive in many eukaryotic cell types and to infect several organs, including the intestine, liver, spleen, brain and placenta (Radoshevich and Cossart, 2017). *Lm* is the etiological agent of listeriosis, a disease characterized by bacteremia, central nervous system infections, spontaneous abortions and perinatal infections (Schlech, 2019; Charlier et al., 2020; Bagatella et al., 2021). Listeriosis leads to the highest number of hospitalizations and case fatality rate of any foodborne zoonosis in Europe (European Food Safety et al., 2021), as well as in the United States (Scallan et al., 2011). These rates have not yet been estimated in other regions of the world, but it is noteworthy that countries in Asia and Africa, as well as Australia, have experienced several listeriosis outbreaks in recent decades, with the largest ever in South Africa (2017-2018; 1060 cases, 216 deaths) (Kaptchouang Tchatchouang et al., 2020). The foods responsible of this massive outbreak were processed meat products, named polony. Foods associated with listeriosis include raw meat and fish, seafood, unpasteurized dairy products and foods contaminated after processing, including soft cheeses and deli meats. In recent years, increased surveillance for *Lm* has shown that this dangerous germ can contaminate a variety of ready-to-eat (RTE) foods, including uncooked vegetables and fresh-cut fruits (Buchanan et al., 2017). For example, in Europe, a multi-country outbreak was attributed to consumption of frozen sweet corn (2015-2018; 53 cases, 10 deaths) (McLauchlin et al., 2021), while recent outbreaks in the United States have been associated with consumption of enoki mushrooms (2020; 36 cases, 4 deaths) and packaged salads (2021; 27 cases, 3 deaths, in two separate outbreaks) (https://www.cdc.gov/listeria/outbreaks/index.html).

The remarkable adaptation of *Lm* to different ecosystems, in the environment (*e.g.,* river, lake and sea water, sewage, soil, plants, decaying plants, fodder, silage, agricultural effluents), a variety of foods, and hosts of diverse species, makes it an important archetype of the “One-Health” approach to infectious diseases, which recognizes the interconnection between human health, animal health and environmental ecosystems. However, there is much to discover about the ecology of *Lm*, in terms of natural reservoirs and mechanisms of transmission. In particular, the potential for asymptomatic carriage of this pathogen has been less explored than its mechanisms of pathogenicity. However, healthy carriers play an important role as reservoirs and dispersion factors of *Lm* in the environment. As early as the 1960s, Gray and Killinger (1966) cited 37 mammalian species from which this pathogen was isolated in the feces (Gray and Killinger, 1966). *Lm* is also found in many species of wild mammals, such as rats, hares, hedgehogs, badgers, deer, moose, otters, raccoons, foxes, bears, boars, monkeys (Giovannini et al., 1988; Inoue et al., 1991; Low and Donachie, 1997; Yoshida et al., 2000; Lyautey et al., 2007; Rothenburger et al., 2015; Weindl et al., 2016; Hydeskov et al., 2019; Parsons et al., 2019; Fredriksson-Ahomaa et al., 2020; Palacios-Gorba et al., 2021) and even aquatic mammals, such as dolphins (Sevellec et al., 2020). *Lm* is also a pathogen of farmed mammals, such as sheep, goats, cattle, pigs, horses and rabbits, and pet animals, such as dogs and cats (Weber et al., 1995; Lyautey et al., 2007; Oevermann et al., 2010; Leclercq et al., 2014; Rothenburger et al., 2015). Oevermann and colleagues recently discussed the current knowledge of listeriosis pathology in naturally susceptible species, focusing on ruminants and the neuro-invasive form of the disease (Bagatella et al., 2021). While most of these studies have looked for *Lm* in sick animals, healthy animals can constitute an important reservoir, releasing the pathogen in the environment through fecal excretion or abortion products. In this regard, it is worth mentioning that *Lm* prevalence can sometimes be as high as 30-40% in the feces of healthy cattle (Weber et al., 1995; Hurtado et al., 2017). Healthy carriage of *Lm* also exists in humans, as described in several studies (Lamont and Postlethwaite, 1986; Muller, 1990; MacGowan et al., 1991; Mascola et al., 1992; Iida et al., 1998; Grif et al., 2001; Grif et al., 2003). However, the techniques used to determine the prevalence of asymptomatic carriage have lacked sensitivity and have generally relied on testing for culturable bacteria in feces. A very recent study has significantly improved the knowledge of the frequency of this carriage, by studying the prevalence of *Lm* DNA in the stool of a French cohort of 900 healthy individuals. The use of PCR amplification of the *Lm* virulence gene *hly* revealed a significant proportion of 10% of asymptomatic *Lm* carriers in this population (Hafner et al., 2021). This study also indicated that metagenomics based on 16S rRNA gene sequencing (*i.e.*, metataxonomics) was less efficient in detecting healthy *Lm* carriers (an order of 5%), whereas full metagenomic studies using shotgun sequencing failed in detecting *Lm* DNA signal in the gut microbiota. This highlights the importance of using sensitive molecular techniques to detect healthy carriers. However, it should be remembered that these molecular biology-based approaches cannot determine whether the detected DNA is from live or dead bacteria.

*Lm* is known to be tolerant to many physical and physicochemical stresses, allowing it to survive in a wide range of environmental conditions (Tasara and Stephan, 2006; Gahan and Hill, 2014; Dorey et al., 2019; Matereke and Okoh, 2020; Quereda et al., 2021; Wiktorczyk-Kapischke et al., 2021). The ability of *Lm* to enter a so-called Viable But Non-Culturable state (VBNC) is also a key factor in the survival of this non-spore-forming bacterium following stress. This property was first described 20 years ago for *Lm* present in water (Besnard et al., 2000) and more recently within human cells (Kortebi et al., 2017), findings which raise the issue of detecting “silent” forms of this pathogen in agriculture, food and asymptomatic hosts (Bierne et al., 2018). Here, we review the state of knowledge on the VBNC status of *Lm*. The various techniques associated with detection of VBNC forms will not be extensively discussed in this review, but are summarized in **Table 1**, and we refer readers to excellent recent reviews on this topic (Kumar and Ghosh, 2019; Dong et al., 2020; Wideman et al., 2021). We apologize to the authors whose work was not cited, due to space constraints.

**Table 1:** Methods to study VBNC *Lm*.

Acronym	Full name	Mechanism of action	References
CTC–DAPI	5- cyano-2,3-ditolyltetrazolium chloride– 4',6-diamidino-2-phenylindole staining	CTC, a redox dye, is reduced to an insoluble fluorescent red CTC-formazan salt by the electron transport chain in actively respiring bacterial cells. The presence of red CTC crystals in bacterial cells is visualized by epifluorescence microscopy. Counterstaining with DAPI, which fluoresces blue, provides contrast and allows enumeration of total bacteria.	Bremer et al., 1998; Besnard et al., 2000; Cappelier et al., 2005; Dreux et al., 2007
DVC	Direct Viable Count	Incubation of the samples in a medium containing a limited level of nutrients and an antibiotic acting as an inhibitor of DNA replication, allows the viable cells to metabolize nutrients and elongate, while blocking their division. After staining with a fluorescent dye, elongated cells are counted by fluorescence microscopy.	Besnard et al., 2000; Besnard et al., 2002; Dreux et al., 2007; Giao and Keevil, 2014; Highmore et al., 2018
DVC –FISH	Direct Viable Count –Fluorescence In Situ Hybridization	This method combines DVC and FISH with a *Lm* fluorescent oligonucleotide probe (usually 16S rRNA) used to visualize bacteria by microscopy.	Moreno et al., 2012
Live/Dead BacLight	Live/Dead BacLight bacterial viability assay	This method discriminates between live and dead bacteria based on membrane integrity, using a dual staining with DNA-intercalating dyes, SYTO 9 green-fluorescent stain and Propidium Iodide (PI) red-fluorescent stain. These dyes differ in their ability to enter bacteria. SYTO 9 enters in all cells, whereas PI only enters bacteria with compromised membranes, causing reduced SYTO 9 fluorescence. With an optimized mixture, dead bacteria will fluoresce red, while live bacteria will fluoresce green.	Dreux et al., 2007; Lindback et al., 2010; Giao and Keevil, 2014; Kortebi et al., 2017; Afari and Hung, 2018; Robben et al., 2019; Gu et al., 2020
Flow cytometry	Flow cytometry	This method estimates the number of viable cells in a heterogeneous population using a flow cytometer. Viable cells are discriminated by fluorescent dyes that either penetrate all cells (*e.g.*, SYTO 9 or SYTO 24) or penetrate only cells with compromised membranes (*e.g.*, PI).	Truchado et al., 2020; Chiara et al., 2021
CFDA	Carboxy-fluorescein diacetate staining	CFDA is a colorless fluorogenic ester that enters bacterial cells through diffusion. CFDA is enzymatically cleaved by esterase enzymes of viable cells yielding a fluorescent probe, which accumulates exclusively in bacteria with intact membranes. This labeling can be used for monitoring viable cells by fluorescence microscopy or flow cytometry.	Olszewska et al., 2015; Arvaniti et al., 2021
ATP	BacTiter-Glo Microbial Cell Viability assay	The method is based on the quantification of ATP. A sample is mixed with a luciferase reagent and the luminescence is recorded in relative light units (RLU) by a luminometer. Data are converted to ATP concentration per cell using a standard curve, and is proportional to the number of viable cells.	Lindback et al., 2010; Robben et al., 2019
qPCR	Quantitative polymerase chain reaction	Amplification of a bacterial DNA fragment by real time polymerase chain reaction. This method indicates presence of bacterial DNA but does not assess viability.	Wery et al., 2006; Klein et al., 2011; Maynaud et al., 2016; Afari and Hung, 2018
RT-PCR	Reverse transcription polymerase chain reaction	Detection of a messenger RNA (mRNA), by PCR amplification of a cDNA sequence synthesized from the mRNA template.	Lindback et al., 2010; Zolfaghari et al., 2020
v-PCR (EMA- or PMA-qPCR)	viability-PCR	Ethidium monoazide (EMA) and propidium monoazide (PMA) are photoreactive DNA-intercalating dyes that, when used in combination with polymerase chain reaction, prevent the DNA of dead bacteria from being amplified. The dyes selectively enter only the compromised cells (PMA being more selective for dead cells than EMA). Exposure to light crosslinks the dye to the DNA and blocks the PCR reaction (generally in qPCR).	Desneux et al., 2016; Overney et al., 2016; Overney et al., 2017; Brauge et al., 2020; Gu et al., 2020; Truchado et al., 2020; Bernardo et al., 2021
Metataxonomics 16S-rRNA	16S rRNA gene sequencing	This method is based on the sequencing of the 16S rRNA gene, which is found in all bacteria and archaea. 16S rRNA gene sequencing is commonly used for identification, classification and quantification of microbes within microbial communities. This method indicates presence of bacterial DNA but does not assess viability.	Pang et al., 2018
Metagenomics	Whole shotgun metagenomic sequencing	Direct sequencing of all the DNA present in a sample. Shotgun sequencing has more power to identify less abundant taxa than 16S rRNA sequencing, but requires in-depth bioinformatics analysis. This method indicates presence of bacterial DNA but does not assess viability.	De Goffau et al., 2019

Only methods cited in this review are listed and ordered as follows: fluorescence-based methods, biochemical methods and molecular biology methods. The reference list provides examples, but is not exhaustive.

## Overview of the VBNC State

Microorganisms colonize an impressive variety of ecological niches and survive a wide range of adverse environmental conditions. The survival strategies employed by bacteria encompass two main mechanisms. The first one is sporulation, which consists of the formation of a specialized cell (or endospore), metabolically inactive and resistant to many environmental perturbations (*e.g.,* heat treatment, high pressure, dehydration, lack of nutrients, antimicrobial agents, UV and γ radiation). When conditions become favorable for regrowth, the spore is able to germinate to regenerate a vegetative bacterium (Setlow and Johnson, 2019). The second mechanism consists of entry into a state of dormancy, allowing non-spore-forming bacteria to survive environmental stress (*e.g.*, nutrient starvation, light exposure, temperature change, osmotic pressure, oxygen concentration, pH), antimicrobial compounds (*e.g.*, disinfectants, antibiotics) or host factors (Kaprelyants et al., 1993). This dormant state is a reversible state of slowed activity, in which a subpopulation of bacterial cells can survive for prolonged periods without cell division (**Table 2**). In bacterial species that transiently lose the ability to grow on standard culture media on which they are known to grow, this is referred to as a VBNC state. Bacteria in the VBNC state (here termed “VBNC bacteria”) fail to multiply and form colonies on plates, while maintaining a slowed metabolic activity. First described in 1982 for Gram-negative bacteria [*Escherichia coli* and *Vibrio cholerae*, (Xu et al., 1982)], the VBNC state is, like sporulation, a reversible state. VBNC bacteria can regain their lost culturability under certain conditions called “resuscitation” (Oliver, 2010; Li et al., 2014; Zhao et al., 2017). Illustrating this property, *Vibrio vulnificus*, an environmental bacterium living in estuarine waters, enters a VBNC state during winter months and regrows during warmer months (Whitesides and Oliver, 1997).

**Table 2:** Definitions of terms used to describe different survival strategies of bacteria to cope with environmental, antimicrobial, or host-derived stress.

Term	Definition
**Sporulation**	When exposed to stress, some bacteria undergo asymmetric cell division to produce a metabolically inactive daughter cell, called a spore. Upon exposure to favorable environmental conditions, a fraction of the spore germinates and initiates rapid growth to restore the population. Sporulation is a specialized development program.
**Dormant state**	Bacteria are in a dormant state when they stop dividing and strongly, or even completely reduce their metabolic activity when exposed to stress. Under specific stimuli, these cells regain their activity and have the ability to divide again. This generic term includes different phenotypes (such as persistence and VBNC states).
**Viable But Non Culturable state**	Bacteria are in a VBNC state when they have transiently lost their ability to grow on routine growth medium on which they were previously able to grow. These bacteria exhibit low but detectable metabolic activity, maintain membrane integrity, express genes and produce proteins at low levels. They cannot be detected by colony forming units (CFU) on standard culture media. Under appropriate conditions, VBNC bacteria regain their ability to be culturable through a process called resuscitation.
**Sublethal state**	Bacteria are in a sublethal state when they have suffered damage to cell structures due to chemical or physical processes, but are not killed and have the ability to repair their damage under appropriate conditions. They can grow on culture media, however none-selective. Sublethally injured cells that remain metabolically active but cannot be resuscitated in culture media may enter the VBNC state.
**Persistence state**	Bacteria are in a persistence state when they enter a state of slow or no growth and are able to survive stress within an otherwise stress-sensitive clonal population. This is a general term that describes an adaptive and reversible process. This term is often associated with persisters.
**Persisters**	This term defines a subpopulation of bacteria that survive antibiotic treatment, without acquiring genetic changes that confer resistance. Persisters are refractory to antibiotic treatment *in vitro* or in the host due to low target activity or low antibiotic uptake.
**Persistent infection**	This clinical term refers to an infection that is not effectively eliminated by the host. Bacteria survive in the host’s tissues for a prolonged period of time despite the host’s immune defenses.
**Latent infection**	This clinical term refers to persistent asymptomatic infection. Latent infection occurs when a microbe persists in a host without disrupting homeostasis sufficiently to cause clinical symptoms or disease. Latency can be beneficial to both the host and the microbe, in a balance where the host avoids progressive damage from interaction with the microbe and the microbe secures a stable niche in which to survive. Latency can be deleterious to the host when a latent infection reactivates, causing symptomatic disease months or years after the initial infection.

It should be noted that, in addition to the VBNC state, other physiological states associated with the survival of bacteria in stressful conditions have been described, such as the sublethal state or the persistent state. The terminology used can be confusing, as the terms sometimes cover overlapping phenotypes, or phenotypes associated with clinical pictures (*i.e.,* persistent infection and latent infection). For clarity, we provide a table defining these terms (**Table 2**). In particular, so-called “persisters” appear following antibiotic treatment, to which they adapt and acquire tolerance to the treatment (Gollan et al., 2019). Some authors propose that persisters and VBNC bacteria are actually part of the same *continuum* in the natural life cycle of microorganisms, between actively growing, dormant and dead cells. VBNC cells would thus be in a deeper dormant state than persister cells (Ayrapetyan et al., 2018). A recent study suggests that sublethal injury is also an initial stage of dormancy that is followed by the VBNC state (Arvaniti et al., 2021). It can be pointed out that VBNC cell subpopulations can also exist naturally in unstressed environments, as the result of a stochastic physiological phenomenon, which leads to a bacterial pool predisposed to resist potential future stresses (Ayrapetyan et al., 2015; Goncalves and De Carvalho, 2016).

Since its discovery in 1982, the VBNC state has been described for more than 100 species, two-thirds of which are pathogenic species (Oliver, 2010; Li et al., 2014; Ramamurthy et al., 2014; Zhao et al., 2017; Dong et al., 2020). This list is constantly growing. In general, bacteria capable of entering the VBNC state have a broad phylogenetic distribution, suggesting that the VBNC state is a general strategy adopted by different genera to survive adverse conditions (Li et al., 2014; Pinto et al., 2015). Currently, the majority of species described as entering the VBNC state are Gram-negative bacteria. Among Gram-positive bacteria, in addition to *Lm*, there are other pathogens of the firmicutes phylum (*e.g.*, *Staphylococcus aureus, Enterococcus faecalis*), and actinobacteria (*e.g.*, *Mycobacterium tuberculosis*). Because of their non-culturable nature and resuscitation capacity, VBNC bacteria are a public health problem. For species responsible for food poisoning, VBNC forms escape quality controls in food industries, as detection of contamination is most often based on methods dependent on bacterial growth in conventional enrichment media.

VBNC bacteria are also suspected of causing asymptomatic infections. However, while the ability to enter a VBNC state has been described for many pathogens, the demonstration that this state exists in the host is virtually undocumented, except in the case of *Mycobacterium tuberculosis* (Mtb). Human infection with Mtb produces an active form of the disease, tuberculosis (TB), one of the top ten causes of death worldwide, as well as an asymptomatic form called latent tuberculosis infection (LTBI) (Gengenbacher and Kaufmann, 2012; Dutta and Karakousis, 2014; Peddireddy et al., 2017). Mathematical models based on immunoassays suggest that a quarter of the world’s population is affected by LTBI, with a risk of reactivation to TB in about 5-10% of cases. In LTBI, Mtb bacilli enter a dormant state characterized by reduced metabolic activity in response to stressful intracellular conditions and activation of the host immune response. The bacteria enter a non-culturable state in the lungs, but also potentially in other organs (Dutta and Karakousis, 2014), without causing any symptoms, a VBNC state termed “latency”. Studies suggest that Mtb bacilli coexist in mixed populations with culturable stages, in a physiological *continuum* from persistence to dormancy (Gengenbacher and Kaufmann, 2012; Dutta and Karakousis, 2014; Gample et al., 2019). Reactivation of TB shows that latent VBNC cells are able to retain or, at least, regain their virulence potential upon resuscitation. Resuscitation factors (Rpfs), initially identified in the bacterium *Micrococcus luteus* (Kaprelyants et al., 1994; Mukamolova et al., 1998) and later in mycobacteria (Mukamolova et al., 2002; Rosser et al., 2017) are involved in this process. Some fatty acid species, adenylate cyclase, transcription factors regulating respiration pathways, and carbohydrate metabolism, also appear to be involved in Mtb resuscitation (Shleeva et al., 2013; Salina et al., 2019).

## Induction of the VBNC State of *Lm* in Water

To our knowledge, the entry of *Lm into* the VBNC state in a natural environment was first suggested by Colburn et al. (1990), following the observation of lower frequency of culturable bacteria detected in marine water samples than in fresh water samples of the same estuary in California (Colburn et al., 1990). Bremer et al. (1998) also hypothesized this after examining the survival of *Lm* in sterilized seawater in the laboratory (Bremer et al., 1998). After 26 days, a fraction of the cell population seemed to be VBNC, as the number of viable cells quantified by measurement of respiratory activity (*i.e.*, CTC-DAPI, **Table 1**) was greater than that determined by traditional colony forming units (CFU) counts on tryptic soy yeast extract agar plates. Besnard et al. (2000) then formally established this capacity of *Lm* to enter the VBNC state (Besnard et al., 2000), by culturing bacteria in a nutrient-free water or “microcosm water” (*i.e.,* sterile filtered water of pH = 6), a medium used to study the VBNC state of *Campylobacter jejuni* (Rollins and Colwell, 1986). Under these conditions, and using Direct Viable Counts (DVC) and CTC-DAPI methods (**Table 1**), these authors showed that two strains of *Lm*, CNL 895807 and ScottA, formed VBNC bacteria after four to six weeks of nutrient deprivation. Furthermore, these forms retained metabolic activity for up to ten weeks. The same team later revealed the influence of inoculum concentration, light exposure, temperature, strain used, pH and NaCl concentration on the efficiency of the phenomenon (Besnard et al., 2002). Thus, a high inoculum concentration decreased the induction of the VBNC state, probably due to a higher nutrient flux within the population. Conversely, higher salt concentration or incubation temperatures stimulated entry into the VBNC state. A strain effect was also found: for the Scott A and CNL 895807 strains, the VBNC state was maintained throughout the experiment, whereas the VBNC state was transient for the LO28 and ATCC 19115 strains. VBNC bacteria induced in microcosm water conserve a metabolic activity, as demonstrated by *de novo* production of ATP and mRNA (Lindback et al., 2010). In the latter study, the quantification of viable bacteria was estimated by the LIVE/DEAD BacLight assay, a dual-staining microscopy method that assesses membrane integrity (**Table 1**).

More recently, a study showed that tap water was also a highly deprived medium in which *Lm* can enter the VBNC state, particularly within biofilms formed on stainless steel surfaces (Giao and Keevil, 2014). Thus, although *Lm* is not known to be a waterborne pathogen, the potential for *Lm* cells to remain on surfaces with only water as a nutrient medium can have a significant impact on public health. If decontamination is insufficient after surface cleaning, *Lm* could remain on food industry surfaces (*e.g.,* floors, walls, workbenches, sinks, refrigerators) and be transferred into food products.

The studies mentioned in this section are listed in **Table 3**.

**Table 3:** VBNC *Lm* in water.

*Lm* strains	Tested conditions	Incubation time	References
Scott A, KM	Filter-sterilized seawater at 12.8 °C or 20.8 °C	20 to 28 days	Bremer et al., 1998
CNL 895807 Scott A ATCC 19115 LO28	Filtered sterilized water adjusted to pH 6.0, incubated at 20°C or 4°C with or without NaCl 7%, with gentle shaking at 100 rpm, 6 log CFU/mL	4 to 6 weeks	Besnard et al., 2000
CNL 895807 Scott A ATCC 19115 LO28	Filtered sterilized water adjusted to pH 6.0, incubated at 20°C or 4°C, with or without NaCl 7%, with gentle shaking at 100 rpm, 8 to 9 log CFU/mL (high inoculum) 6 log CFU/mL (low inoculum)	Up to 12 months 28-80 days (9-28 days under natural sun light)	Besnard et al., 2002
Scott A EGDe 14 strains from different origins	Filtered, autoclaved MQ water adjusted to pH 6.0, incubated at 4°C, with gentle shaking at 100 rpm, 7 log CFU/mL	5 to 12 weeks	Lindback et al., 2010
NCTC 13372	Biofilm on stainless steel coupons in tap water, incubated at different temperatures, 7 log CFU/mL	24 and 48 hours	Giao and Keevil, 2014

Studies are listed by publication date in the References column.

## Induction of the VBNC State of *Lm* Under Agro-Industrial Conditions and in Foods

In addition to water, all ecosystems in the agricultural and food industries can be potentially impacted by VBNC *Lm* contamination. Because of the health risks of such contamination, many studies have addressed this problem by modeling the contamination by VBNC *Lm* in reconstituted systems (also called bacterial microcosms). The main technical difficulty is to correctly differentiate dead from VBNC cells in complex matrices. Works have examined the persistence of *Lm* in urban or agricultural effluents, after inoculation of bacteria into sewage sludge (Wery et al., 2006), cattle manure (Klein et al., 2011), or digestates from agricultural waste methanizers (Maynaud et al., 2016). In these studies, comparison of viable bacterial counts estimated by amplification of a bacterial gene by quantitative real-time PCR (qPCR) (**Table 1**), to that of culturable bacteria by CFU, showed a slower decline of *Lm* cells counted by PCR than by CFU, suggesting the presence of non-culturable cells in these microcosms. However, qPCR does not distinguish DNA of non-culturable bacteria from dead bacteria, generating a risk of overestimating the number of VBNC bacteria. Accurate quantification of only viable bacteria has been improved by a so-called “viability-PCR” (v-PCR), a method combining the use of an amplification inhibitor, such as propidium monoazide (PMA) or ethidium monoazide (EMA), and qPCR (PMA-qPCR; EMA-qPCR, **Table 1**). In particular, PMA is a DNA intercalant which enters into cells with compromised membrane integrity and blocks amplification of their DNA. Treating bacterial cells with PMA, prior to DNA extraction, therefore allows for more selective detection of viable bacteria (although there remains a bias for DNA amplification from dead cells that would have retained an intact membrane) (Dong et al., 2020; Fleischmann et al., 2021; Wideman et al., 2021). This technique provided strong evidence for the presence of VBNC *Lm* in pig manure (Desneux et al., 2016), as well as in food environments (see below and **Table 1**).

Other studies reported induction of the VBNC state of *Lm* on the surface of plants, including parsley (Dreux et al., 2007) and spinach leaves (Highmore et al., 2018). Indeed, *Lm* has evolved mechanisms to colonize plants (Truong et al., 2021). The possibility that conventional culture-based counts underestimate the number of viable *Lm* on plant products must be considered in the context of cross-contamination risks, when a contaminated product is washed and pathogens are transferred from the contaminated product to water and then from water to clean product. Two studies looked at this problem, investigating whether process wash water (PWW) could be a vector for VBNC contamination. *Lm* contamination of a PWW was modeled in the laboratory, either by inoculating a PWW of shredded lettuce with a cocktail of six *Lm* strains (Truchado et al., 2020) or spinach and lettuce rinse waters with a cocktail of three *Lm* strains (Gu et al., 2020). Potential VBNC were detected in these wastewaters by the PMA-qPCR method or viability microscopy with the BacLight assay **(** **Table 1**). Importantly, in both cases, exposure to sanitizers increased the formation of unculturable viable cells (see next section).

With respect to foods, there is ample evidence for the presence of VBNC in food matrices or food processing environments. Olszewska et al. (2015) studied the fate of *Lm* after bacterial inoculation of long-ripened hard cheese samples from retail market, left in different packaging conditions for up to 90 days at 6°C (Olszewska et al., 2015). As an approach to assess bacterial viability in complex matrices, these authors used direct epifluorescence filtration, based on fluorescence staining with carboxyfluorescein diacetate (CFDA), which allows tracing bacteria that exhibit metabolic activity (**Table 1**). Their results showed significant discrepancies between CFDA and CFU numbers at each sampling point during cheese storage, regardless of packaging conditions, indicating the existence of VBNC cells. Overney et al. (2016) studied the behavior of *Lm* grown in two food contaminants, smoked salmon juice or meat exudate, by quantifying total cells by qPCR, viable cells by PMA-qPCR, and culturable cells by CFU counting (Overney et al., 2016). Meat exudate appeared to be a much more stressful medium than smoked salmon juice, and generated VBNC cells. These authors proposed that these differences between meat exudate and smoked salmon juice could be explained by the difference in pH (5.4 for meat exudate, 6.2 for smoked salmon juice), which is an environmental stress. Importantly, there is evidence for the existence of VBNC forms, no longer in laboratory models, but directly in food samples. With a DVC method coupled with FISH using a 16S rRNA probe (**Table 1**), Moreno et al. (2012) suggested the existence of VBNC *Lm* in RTE vegetable samples (Moreno et al., 2012). Recently, a study using PMA-qPCR provided strong arguments for the existence of VBNC *Lm* in RTE salads (Bernardo et al., 2021). Sources of product contamination can come directly from surfaces in the production environment. By directly sampling critical areas of four seafood plants, over eight months, Brauge et al. (2020) detected VBNC *Lm*, primarily after cleaning and disinfection operations (Brauge et al., 2020), highlighting the fact that all industrial disinfectants can fail to inactivate *Lm* cells on inert surfaces, as discussed below.

The studies mentioned in this section are listed in **Table 4**.

**Table 4:** VBNC *Lm* in agri-food industrial conditions.

*Lm* strains	Tested conditions	Incubation time	References
Scott A	Sewage sludge, at 20°C, 6 log CFU/mL	2, 5, 9 and 17 days	Wery et al., 2006
EGDe, EGDe-GFP LmP60	Parsley leaves under low relative humidity (47–69%) 7 log CFU/mL (high inoculum) 4.5 log CFU/mL (low inoculum)	2 and 15 days	Dreux et al., 2007
Endogenous strains	Cattle feedlot manures and compost, stored at 4 °C in the dark	112 days	Klein et al., 2011
Endogenous strains	Samples of RTE foods of vegetal origin from market		Moreno et al., 2012
ATCC19112	Cheese samples of long-ripened hard cheese, at 6°C, 6-7 log CFU/g	90 days	Olszewska et al., 2015
A strain isolated from pig manure	Nine piggery effluents		Desneux et al., 2016
CCL 500	*Lm* cultured in smoked salmon juice or meat exudate, at 25°C, 3 log CFU/mL	48 hours	Overney et al., 2016
Rifampicin-resistant mutant obtained from CIP110870 (isolated from pig manure)	Digestates from agricultural biogas plants, at 24°C, 6-7 log CFU/mL	40 days	Maynaud et al., 2016
Scott A-GFP	Spinach leaves washed in chlorinated water, 6 log CFU/mL	2 minutes	Highmore et al., 2018
3-strain cocktail (FS2025, FS2030, FS2061)	Rinse water of spinach or romaine lettuce leaves (untreated or chlorinated or peracetic-treated water), 6 log CFU/mL	30 seconds	Gu et al., 2020
6-strain cocktail	Chlorine-treated process wash water from washing shredded lettuce, 5 log CFU/mL	30 seconds + 1 and 3 hours	Truchado et al., 2020
Endogenous strains	Environmental samples collected in smoked salmon processing plants		Brauge et al., 2020
Endogenous strains	Samples of ready-to-eat (RTE) salad, incubated at 4, 12 or 16°C	Up to 8 days	Bernardo et al., 2021

Studies are listed by publication date in the References column.

## Induction of the VBNC State of *Lm* by Exposure to Antimicrobial Treatments and in Biofilms

The eradication of microorganisms in food processing plants, hospitals and domestic environments is most often done through disinfectants, while preservatives are used to maintain the microbiological quality of food, and antibiotics remain the first-line treatment for bacterial infections. However, many of these antimicrobial agents are not very effective on VBNC bacteria, which not only have a slower metabolism, but a different morphology and composition, than vegetative bacteria, making them more resistant to physico-chemical stress. (Li et al., 2014; Dong et al., 2020). Additionally, and importantly, as entering a VBNC state is a physiological adaptation to stress, these antimicrobial treatments may stimulate formation of VBNC bacteria. In the case of *Lm*, an initial study suggested that exposure of bacteria to potassium sorbate could promote the formation of VBNC *Lm* at 37°C (Cunningham et al., 2009). Later, chlorinated treatments were associated with the appearance of VBNC forms, both in fresh produce (Highmore et al., 2018) and in wastewater (Truchado et al., 2020). This is of particular concern because chlorine is the most commonly used sanitizer in industry and its efficacy is typically assessed using culturable bacterial counts (López-Gálvez et al., 2019). It is worth noting that bacterial adaptation to chlorine-containing disinfectants induces physicochemical and morphological changes in *Lm* described for other species entering the VBNC state, in particular a transition from the typical rod shape of *Lm* bacilli to a coccoid shape (Gao and Liu, 2014). Treatments with lower concentrations of chlorine or benzalkonium chloride also show induction of VBNC *Lm* (Afari and Hung, 2018; Noll et al., 2020; Truchado et al., 2020). VBNC *Lm* also emerged in presence of other antimicrobial agents, such as surfactants (Robben et al., 2018), preservatives (Zolfaghari et al., 2020), essential oils (De Medeiros Barbosa et al., 2020) or peracetic acid (Gu et al., 2020; Arvaniti et al., 2021). Chiara et al. (2021) interestingly showed that the origin of the bacterial cells has a significant impact on the viability and culturability of *Lm* against antimicrobial treatments. Thus, mild heat treatment combined or not with terpenoids mainly affected cell culturability (assessed by CFU count) rather than viability (assessed by flow cytometry, **Table 1**) (Chiara et al., 2021).

Some new technologies to ensure the preservation and safety of RTE without the use of disinfectant molecules, or heating treatment, may also induce a loss of cultivability of *Lm* while maintaining a viable subpopulation. This is suggested by experiments conducted not directly on *Lm*, but on the closely related non-pathogenic species *Listeria innocua*, to evaluate a pulsed light treatment. A significant discrepancy was observed between the number of conventional CFUs and the various viability staining parameters, suggesting that a significant proportion of bacteria shifted to the VBNC state after pulsed light treatment. The loss of cultivability was associated with oxidative stress and DNA damages, rather than cell membrane disruption or inactivation of intracellular enzymes (Kramer and Muranyi, 2014). It is important to mention that not only may VBNC *Lm* be induced by industrial treatment, but also tolerate higher concentrations of antimicrobials after adaptation to a first treatment (Noll et al., 2020) or upon a combination of treatments (Robben et al., 2018). In addition, VBNC forms induced by industrial cleaning products can become tolerant to antibiotics, particularly those used to treat listeriosis, such as ampicillin and gentamicin (Robben et al., 2019; Noll et al., 2020).

*Lm* like many other bacterial species can form biofilms, which also counteract general cleaning and disinfection procedures and make it difficult to remove this pathogen from food processing surfaces (Bridier et al., 2015). It is of note that *Lm* can enter a VBNC state within a biofilm (Chae and Schraft, 2001; Giao and Keevil, 2014; Brauge et al., 2018). Highmore et al. (2018) showed that VBNC *Lm* adhering to the surface of spinach leaves are present at junctions and stomata, a location favoring biofilm formation on the leaf surface. Despite the protective effect of the biofilm, chlorine exposure manages to induce the VBNC state within the biofilm (Highmore et al., 2018). The molecular characteristics of the bacterial surface appear to play a role in the efficiency to enter the VBNC state. For example, Brauge et al. (2018) showed that *Lm* mutants lacking N-acetylglucosamine in wall teichoic acids generate a higher population of VBNC in a biofilm (Brauge et al., 2018). The same team thereafter revealed that exposure of *Lm* biofilms on stainless steel or polyvinyl chloride to quaternary ammonium and hydrogen peroxide disinfectants reduced cultivable populations and induced VBNC populations (Brauge et al., 2020). Similarly, Overney et al. (2017) showed induction of the VBNC state in *Lm* biofilms formed on stainless steel after treatment with a chlorinated alkaline cleaner or quaternary ammonium disinfectant (Overney et al., 2017). Of note, in the presence of disinfectants, the difference between initial and final cell number was not significantly different when bacteria were grown in the presence of smoked salmon juice or meat exudate (Overney et al., 2017), which contrasts with the effect of the food soil itself (Overney et al., 2016) and further highlights the impact of stress induced by cleaning procedures on VBNC formation.

The studies mentioned in this section are listed in **Table 5**.

**Table 5:** VBNC *Lm* induced by antimicrobials and/or in biofilms.

*Lm* strains	Tested conditions	Incubation time	References
Murray 7148	Biofilm on glass slides at 37°C, 7 log CFU/mL	Up to 10 days	Chae and Schraft, 2001
10403S	BHI adjusted to pH 4.0 with HCl, incubated at 37°C in the presence or absence of 50 mM K sorbate	24 hours	Cunningham et al., 2009
NCTC 13372	Biofilm on stainless steel coupons in tap water, incubated at different temperatures, 7 log CFU/mL	24 hours	Giao and Keevil, 2014
*Listeria innocua* DSM 20649	Pulsed light treatment (on a heat-stable polysaccharide gel surface with one single intense pulse), 6 to 7 log CFU/cm^2^	one pulse	Kramer and Muranyi, 2014
9 ATCC strains from different serotypes	Chloramine-T or sodium hypochlorite in TSB at 37°C, 6 log CFU/mL	20 hours	Gao and Liu, 2014
LO28 EGDe	Biofilm on stainless steel surfaces, with different cleaning and disinfection procedures using Topax, a chlorinated alkaline cleaner or Triquart, a quaternary ammonium-based disinfectant, 8 log CFU/coupon	4 days	Overney et al., 2017
EGDe EGDe ∆l*mo2549* EGDe ∆l*mo2550* DSS 1130 BFA2	Biofilm on stainless steel coupons at 30°C, 7 log CFU/mL	48 hours	Brauge et al., 2018
Scott A-GFP	Spinach leaves washed in chlorinated water, 6 log CFU/mL	2 minutes	Highmore et al, 2018
Cocktail of five nalidixic acid-adapted strains (obtained from Scott A, LCDC-81-861, F8027, H7750 and G3990 strains)	Electrolyzed oxidizing (EO) water (pH 2.3), after low free chlorine concentration treatments, 6 log CFU/mL	1 and 5 minutes	Afari and Hung, 2018
EGDe	Combinational effect of surfactants and salts at room temperature, 9 log CFU/mL	1 and 24 hours	Robben et al., 2018
3253 EGDe QC1 Scott A	11% Lutensol XP30 + 1 M MgCl2, at room temperature, 9 log CFU/mL Susceptibility tests to antibiotics: ampicillin, ciprofloxacin, gentamicin, imipenem Test of quaternary ammonium compounds (*e.g.*, Benzalkonium chloride) and preservatives (bronopol, sodium azide)	24 hours	Robben et al., 2019
3-strain cocktail (FS2025, FS2030, FS2061)	Rinse water of spinach or romaine lettuce leaves (untreated or chlorinated or peracetic-treated water), 6 log CFU/mL	30 seconds	Gu et al., 2020
SLCC2540s	- Adaptation to benzalkonium chloride - Susceptibility tests to antibiotics: ampicillin, benzylpenicillin, ceftriaxone, ciprofloxacin, daptomycin, erythromycin, gentamicin, linezolid, meropenem, rifampicin, tetracycline, tigecycline, trimethoprim/sulfamethoxazole, vancomycin, at 37°C, 8.2 log CFU/mL	24 hours	Noll et al., 2020
Cocktail of three strains (ATCC 7644, ATCC 19112, ATCC 19117)	Essential oils in meat-based broth and PBS at 30°C, 7 log CFU/mL	60 to 180 minutes	de Medeiros Barbosa et al., 2020
ATCC 19115	Distilled water, with NaCl 30% at 4°C, 7 log CFU/mL	27 days	Zolfaghari et al., 2020
6-strain cocktail	Chlorine-treated process wash water from washing shredded lettuce, 5 log CFU/mL	30 seconds + 1 and 3 hours	Truchado et al., 2020
Lm1 Endogenous strains	- Single species and mixed biofilms grown on stainless steel and PVC after quaternary ammonium compound (QA) and hydrogen peroxide (HP) treatment, 9 log CFU/mL - Environmental samples collected in smoked salmon processing plants before cleaning and disinfection (C&D) operations	20 minutes	Brauge et al., 2020
Scott A	Non-treated (control) or treated bacteria (mild heating (55°C), for 30 min combined or not with terpenoids) and stored at 4°C, followed by inoculation on Gorgonzola rind, 4.6-4.8 log CFU/g	3 and 7 days	Chiara et al., 2021
Scott A	Exposure to peracetic acid at 40 ppm, at 20°C, 9 log CFU/mL	3 hours	Arvaniti et al., 2021

Studies are listed by publication date in the References column.

## Induction of the VBNC State of *Lm* in the Host

**Figure 1:**
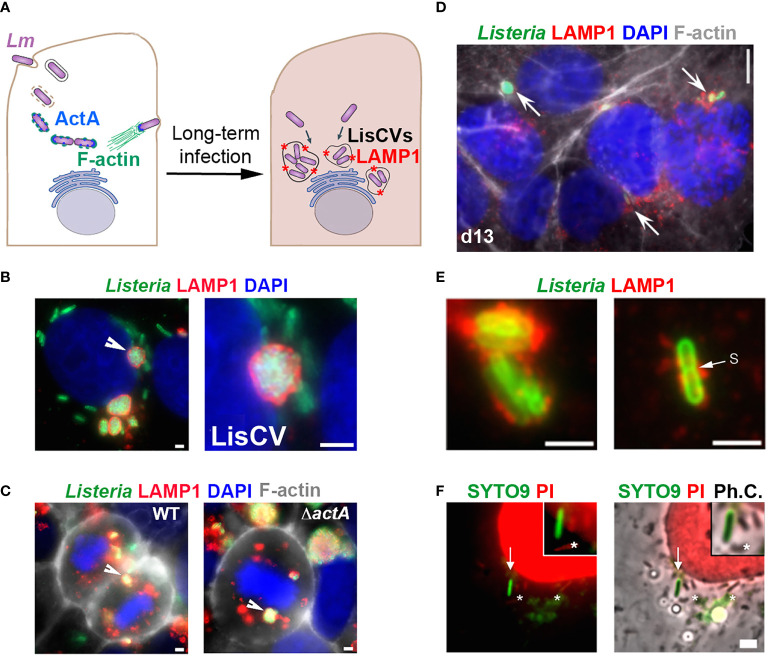
Evidence for an intracellular VBNC state of *L. monocytogenes* (adapted from (Kortebi et al., 2017). **(A)** Simplified diagram of the different phases of the intracellular life of *Lm* in epithelial cells. Bacteria are internalized into the host cell and are contained in an entry vacuole. After escape into the cytosol, bacteria multiply and produce ActA, which allows them to polymerize actin (F-actin), move into the infected cell, and spread to adjacent cells (not shown). After a few days of infection, cytosolic bacteria cease to produce ActA and are captured in membrane compartments, forming *Listeria-containing vacuoles* (LisCVs) marked with LAMP1 (represented by red stars). In these acidic vacuoles, a subpopulation of bacteria can resist degradation and multiply slowly, up to entry into dormancy. **(B, C)** Observation of LisCVs by epifluorescence microscopy in human placental JEG3 cells infected for 3 days with *Lm*. LisCVs labelled with LAMP1 are in red, *Listeria* in green, DNA (stained with DAPI) in blue, and F-actin in white. **(B)** A cell contains several perinuclear LisCVs. The arrow indicates a LisCV shown at higher magnification in the image on the right. Bars: 2 μm. **(C)** LisCVs are present in mitotic cells infected with either the wild-type *Lm* strain (WT, left) or a Δ*actA* mutant (right). Bars: 2 μm. White arrows indicate representative LisCVs. **(D–F)** Subculturing of JEG3 cells infected with Δ*actA* bacteria leads to VBNC bacteria. **(D)** Cells infected for 3 days were subcultured and propagated until day 13 (d13) and stained as in **(C)**. Intracellular bacteria in LAMP1-positive compartments are indicated by arrows. At the same time, plating of infected cell lysates onto agar plates produces no colony (not shown). **(E)** High magnification images of non-culturable LAMP1-positive bacteria at d13. A bacterium with a division septum (s) is shown on the right. Bar: 5 μm. **(F)** JEG3 cells infected with non-culturable bacteria were permeabilized with 0.1% Triton X-100 and stained with SYTO9 and PI (see BacLight assay, **Table 1**). Intact VBNC bacteria are stained green (arrow), while damaged bacteria (*) and nuclei are stained red. “Ph.C.”: phase contrast. Bar: 1 μm. The top right squared images show higher magnifications, with one bacterium with an intact membrane (green) and three bacteria with a compromised membrane (red).

The hypothesis that *Lm* can enter a VBNC state in the host, particularly in an intracellular phase, has emerged from work in human epithelial cell infection models. The study of prolonged *Lm* infection in hepatocytes or trophoblast cells revealed a change in the pathogen’s intracellular lifestyle, between the well-known active phases of cytosolic replication and intercellular dissemination (Radoshevich and Cossart, 2017) and a novel so-called “persistence” phase, in which the bacteria enter into dormancy within vacuoles, named LisCVs (*Listeria*-containing-vacuoles) (Kortebi et al., 2017). Thus, after a few days of infection, cytosolic bacteria cease to produce the actin polymerization factor ActA and are captured by intracellular membranes in an a xenophagy-like process, forming acidic vacuoles marked by the lysosomal protein LAMP1 (**Figure 1A**). In these single-membrane perinuclear compartments (**Figure 1B**), a bacterial subpopulation is degraded, while a majority population resists stress and enters a state of slowed growth. LisCVs remain intact in dividing cells during mitosis (**Figure 1C**), in the same way that lysosomes do not disintegrate during mitosis, but are partitioned as separate vesicles. This ability of LisCVs to segregate in daughter cells could be a way for dormant intracellular *Lm* to spread during epithelial tissue renewal.

Several evidences strongly suggest that a subpopulation of bacteria enters the VBNC state in LisCVs. First, after three days of infection, the number of intracellular bacteria quantified by microscopy is much higher than that determined by the classical CFU method (Kortebi et al., 2017). Second, the Baclight Live/Dead technique (**Table 1**) indicates that two-thirds of the vacuolar bacteria retain their membrane integrity. Additionally, when the *actA* gene is kept inactive, vacuolar *Listeria* all cease to be culturable on agar media and can propagate during host cell mitosis (**Figure 1C**), without being degraded by intracellular host defense mechanisms, such as autophagy (Kortebi et al., 2017). After several passages, parasitized host cells still harbor non-culturable bacteria in LAMP1-positive compartments (**Figure 1D**). A few bacteria exhibit a division septum (**Figure 1E**) and several retain membrane integrity, as indicated by the Baclight assay (**Figure 1F**), which is an indication of viability. Inhibition of ActA production is necessary to maintain the VBNC state during long-term infection, as this phenotype is reversible upon re-expression of the *actA* gene in an Δ*actA* mutant. Of note, reactivation of a subpopulation of vacuolar dormant bacteria was observed randomly during host cell passages. This re-growth process occurs by a stimulus not identified to date (Kortebi et al., 2017).

Overall, these results invite to consider the possibility that *Lm* may generate intracellular VBNC forms that might not be detected in tissue samples by bacterial growth-based diagnostic tests. The capacity to persist in intracellular vacuoles could also be involved in *Lm* escape from antibiotic therapy. We found that a high-dose of gentamicin cleared cytosolic *Lm* but not vacuolar *Lm* (Kortebi et al., 2017). LisCV could thus be a niche where *Lm* is protected from gentamicin and other antibiotics, similar to the aminoglycoside-tolerant small colony variants of *Staphylococcus aureus* (Mitchell et al., 2010), or the extracellular persisters of *Lm*, which emerge during bacterial growth in antibiotic-supplemented broth (Knudsen et al., 2013). In addition to antibiotic tolerance, it is possible that VBNC *Lm*-bearing host cells are not recognized and destroyed efficiently by immune cells. Related to this idea, we recently demonstrated that the vacuolar persistence phase of *Lm* in hepatocytes coincided with transcriptional inhibition of genes encoding acute phase proteins, which are important effectors of innate immunity (Descoeudres et al., 2021).

The existence of VBNC *Lm in vivo* remains to be formally demonstrated, but some studies support the hypothesis. As early as 1948, while trying to isolate the germs responsible for bovine listeriosis, Gray et al. (1948) reported the non-culturability of *Lm* (Gray et al., 1948). Thus, the plating on tryptose agar of crushed brains of animals presenting clinical signs of listeriosis revealed no, or very few, bacterial colonies after 24 hours at 37°C. Surprisingly, re-spreading the same samples several months later, after storage in a refrigerator at 4°C, generated numerous colonies. As Gray and Killinger, (1966) reported in a well-documented review, this phenomenon has been described in several other studies showing that after natural or laboratory infections, culture from tissue suspension or swabs did not always reveal the presence of *Lm* (Gray and Killinger, 1966). It was necessary to refrigerate infected tissue for several weeks or months at low temperatures to detect the bacteria. Since that time, culture media for isolating *Lm* have been greatly improved, allowing more efficient culturability of the pathogen. However, these early observations are still intriguing and highlight the possibility that a listerial infection may be overlooked simply because the bacteria were not successfully isolated on the first culture attempt. Furthermore, the mechanism of the culturability enhancement effect at 4°C described by Gray et al. (1948) is not understood, and it is surprising that this phenomenon has not been further investigated. Recently, however, the existence of VBNC *Lm* has been evoked in a model of maternal-neonatal listeriosis in macaque monkeys, based on inconsistent results between CFU counts, which were sometimes negative on blood agar plates, while microscopy data showed Gram-positive rods in tissues (Wolfe et al., 2019). This model is of particular interest because the placentation, physiology, and immunology of pregnancy in the macaque is similar to that of the human species. A notable finding of this study is that some female macaques infected with a low inoculum of *Lm* were able to carry their pregnancies to term with no apparent signs of disease in either the mother or the neonate, whereas histological and cytokine examination of tissues after surgical removal revealed intrauterine signs of inflammation. This suggests the low-level impact of asymptomatic listeriosis, the subsequent consequences of which on the child are not known (Wolfe et al., 2019).

Molecular biology techniques based on PCR or DNA sequencing are currently the most effective in suggesting the presence of VBNC forms in host. From this perspective, we can cite two recent studies highlighting incidents of unusual detection of *Lm* DNA by metataxonomics or metagenomics approaches (**Table 1**). The first detected *Lm* DNA among all bacterial species identified in milk directly collected from the udder of healthy cows in a sterile manner (Pang et al., 2018). The second studied the placenta microbiome of women with gestational or delivery problems compared to women with normal pregnancies. The results revealed the presence of *Lm* DNA in one placenta, that of a mother whose pregnancy resulted in a premature birth but was not diagnosed for listeriosis (De Goffau et al., 2019). While these data do not demonstrate the existence of VBNC *Lm in vivo*, they do raise the importance of developing sensitive techniques to screen for *Lm* in at-risk humans, as well as in potentially healthy animals carrying the pathogen (see next section). As mentioned above, a PCR-based approach was much more effective in detecting *Lm* in the feces of healthy humans than metataxonomics or metagenomics (Hafner et al., 2021). Therefore, PCR-based approaches should be preferred to detect VBNC bacteria. However, development of sensitive viability estimation methods is required to determine the physiological status of the bacteria present in the tested samples.

## Resuscitation and Virulence Potential of VBNC *Lm*

VBNC forms participate in the dispersal and silent transmission of pathogens, but their ability to cause disease requires returning to an active state of virulence. This involves a conversion of dormant, non-culturable cells to an active, culturable state, a process termed “resuscitation” (Roszak et al., 1984; Baffone et al., 2006; Oliver, 2010; Ayrapetyan et al., 2018). Resuscitation is not synonymous with regrowth: a bacterium can be resurrected without dividing, and it is only after having completed the molecular program of exit from dormancy that it can divide on a nutrient-rich medium. To achieve the resuscitation of VBNC cells various physicochemical methods have been tested on different bacterial species, such as temperature shifts or supplementation with various nutrients or metabolites (Pinto et al., 2011; Li et al., 2014; Dong et al., 2020). However, in the case of *Lm*, most of these methods have failed to promote the resuscitation of VBNC forms (Lindback et al., 2010; Afari and Hung, 2018; Robben et al., 2018; Gu et al., 2020). The relative humidity of the environment could play a role for *Lm* exit from the VBNC state on leaf surfaces: resuscitation of VBNC *Lm* was observed when the relative humidity was close to 100%, but not in a dry environment (Dreux et al., 2007). Another study showed *Lm* cells heated in presence or absence of terpenoids recovered a partial culturability when inoculated on Gorgonzola rind, suggesting that VBNC cells can resuscitate under favorable conditions on food, such as higher availability of nutrients and other factors intrinsic to this food matrix (pH, water activity, relative humidity) (Chiara et al., 2021). However, these data did not exclude that the phenomenon resulted from a subpopulation of bacteria that remained culturable.

The possibility of VBNC *Lm* resuscitation in the host was first questioned by the experiments of Cappelier et al. (2005) showing that *Lm* VBNC remained avirulent *in vitro*, in tests of infection of human colon cells (HT-29), and *in vivo*, in a murine listeriosis model (Cappelier et al., 2005). Nevertheless, the same authors later observed that *Listeria* VBNC could resuscitate after incubation for two to six days in an embryonated chicken egg and regain their pathogenicity (Cappelier et al., 2007). Importantly, embryonated eggs allowed better recovery than non-embryonated eggs, suggesting that the substance involved in recovery is located in the chick embryo. Experiments in the worm *Caenorhabditis elegans* also support the hypothesis of VBNC *Lm* resuscitation in the host, through the observation of bacterial colonization of the intestinal tract after inoculation of VBNC forms (Highmore et al., 2018). Furthermore, in models of VBNC *Lm* formation in human epithelial cells, data suggest that subpopulations of unculturable *Lm* have the ability to return to an active growth stage, upon passages of infected host cells (Kortebi et al., 2017).

As previously mentioned, a family of proteins named Rpfs are involved in the resuscitation mechanism of *Micrococcus luteus* (Kaprelyants et al., 1994; Mukamolova et al., 1998) and Mycobacteria (Mukamolova et al., 2002; Rosser et al., 2017). *Lm* encodes two Rpf orthologs: Lmo0186 and Lmo2522 (Ravagnani et al., 2005; Pinto et al., 2013). Like the Rpf prototypes (Mukamolova et al., 2006; Telkov et al., 2006), *Lm* Rpf-like proteins have a lytic transglycosylase domain predicted to cleave glycosidic bonds in the peptidoglycan (PG). In agreement with this, recombinant Lmo0186 and Lmo2522 proteins have a muralytic activity on crude preparations of *Lm* cell walls, as well as on (NAG)3 MUF, an artificial polymer commonly used as a substrate for lysozyme (Pinto et al., 2013). However, while these enzymes demonstrate bacterial growth stimulating properties in minimal media, data indicating that they resuscitate VBNC *Lm* are still missing. More generally, if the muralytic activities of Rpfs are known to play a role in bacterial division, the functional mechanism of resuscitation remains to be clarified. Several hypotheses are proposed. An Rpf could act as a cytokine that, after being secreted by culturable cells, activates a receptor on the surface of VBNC cells and triggers the bacterial resuscitation process (Panutdaporn et al., 2006). Alternatively, it is not Rpf directly, but second messengers derived from PG lysis, that activate the regulatory cascade necessary for the resumption of growth, both of the local cell and, through diffusion, of adjacent bacteria (Nikitushkin et al., 2013). Another hypothesis proposes that an Rpf may be required to cleave a modified PG and release physical constraints, allowing cell growth to resume. This hypothesis is based on the observation that, in some bacterial species, the PG of VBNC cells undergoes increased total cross-linking and modifications, such as O-acetylation, which create a physical barrier for cell growth (Signoretto et al., 2000; Signoretto et al., 2002; Pfeffer et al., 2006). These hypotheses are not mutually exclusive. In any case, the conditions that trigger the expression of genes encoding Rpfs remain to be elucidated.

## The Problem of VBNC *Lm* in At-Risk Persons: Pregnant Women or Immunocompromised People

The asymptomatic phase of invasive listeriosis (*i.e.,* the incubation period) can be long, particularly when associated with pregnancy [up to 3 months, (Goulet et al., 2013) (Angelo et al., 2016)], but the mechanisms associated with this asymptomatic phase are poorly understood. Furthermore, in maternal-neonatal (MN) listeriosis, women usually present with mild febrile symptoms or may even be asymptomatic (Charlier et al., 2020) and blood culture remains negative in about half of the cases tested (Charlier et al., 2017). This raises the question whether detection methods based on the culturability of the pathogen in pregnant women could miss VBNC *Lm*. This problem of screening could be particularly critical, should *Lm* cause early miscarriages. Currently, the dogma is that MN listeriosis is associated with late stages of pregnancy (*i.e.,* second or third trimester) (Charlier et al., 2020) but miscarriages occurring before 14 weeks of amenorrhea are generally not tested for *Lm*. Early spontaneous abortion associated with listeriosis may therefore be underdiagnosed. In support of this hypothesis, in a recent study examining 43 patients with spontaneous abortions, two vaginal swabs were positive for *Lm* by PCR test, of which one was negative for *Lm* by culture on agar plates (Fall et al., 2020). In addition to under-diagnosis of non-culturable *Lm*, reactivation of these dormant bacteria after a silent phase should also be considered. Data from the 1960s suggested that *Lm* present in the reproductive tract could reactivate during pregnancy and cause early fetal loss (Rappaport et al., 1960; Dungal, 1961; Gray et al., 1963; Gray and Killinger, 1966). Experimental work in a laboratory model of feto-placental listeriosis in rabbits supported this hypothesis. After inoculation with *Lm*, some rabbits showed spontaneous abortions with isolation of the bacterium, and after a novel gestation without further inoculation of the pathogen, could show reproductive failure during the following months (Gray et al., 1955). These observations opened the hypothesis that *Lm* could persist in the reproductive tract for a relatively long period of time without signs of disease, and trigger infection in subsequent pregnancies. This has however not been investigated further. As mentioned previously, recent data suggest that VBNC *Lm* might be present in the fetoplacental unit in a listeriosis model in macaques (Wolfe et al., 2019). It seems important to reconsider the notion of persistence of unculturable *Lm* in the female genital tract and subsequently in recurrent pregnancy losses (RPL). RPL is defined as three or more consecutive pregnancy losses before 20 weeks’ gestation. Its etiology is often unclear and may be multifactorial (Pandey et al., 2005). A role for latent listeriosis in this condition has been suggested (Rappaport et al., 1960; Dungal, 1961; Romana et al., 1989), but more research is needed to validate or refute this hypothesis.

Outside of pregnancy cases, *Lm* is so far not described as a pathogen causing subclinical silent infections, but we cannot exclude that this bacterium hides in the organism for long periods of time, especially in a VBNC state within an intracellular vacuole (Bierne et al., 2018), as has been described for Mtb. High-risk factors for sporadic non-perinatal listeriosis are primarily immunosuppressive diseases (Friesema et al., 2015; Charlier et al., 2017). The awakening of dormant intracellular *Lm*, long after ingestion of contaminated food, should be investigated as a potential cause of sporadic listeriosis cases. The existence of a dormant subpopulation of *Lm* could also be implicated in cases of recurrent listeriosis (McLauchlin et al., 1991; Sauders et al., 2001). For example, in a cancer immunotherapy approach using an attenuated *Lm* strain as a vaccine vector, the inoculated strain led to bacteremia 31 months after the initial injection, despite the administration of antibiotic therapy in the patient (Fares et al., 2018).This strain was never detected in the patient’s medical follow-up, as the blood culture remained negative. This phenomenon remains unexplained, but the investigators point to the fact that the patient had prostheses that may favor the formation of a *Lm* biofilm. While *Lm* is rarely implicated in prosthetic joint infections, there are now increasing reports of cases, particularly in immunocompromised individuals (Charlier et al., 2012) and chronic infections with *Lm* linked to these prostheses have been reported (Kleemann et al., 2009; Muchaamba et al., 2020; Hutchins et al., 2021). It is possible that the molecular mechanisms of fracture repair cause a momentary suppression of local immunity, while the development of a biofilm on the joint offers *Lm* protection from antibiotic treatment (Fares et al., 2018). In addition, the settlement of *Lm* in the VBNC state, either in an extracellular biofilm or within intracellular vacuoles, could contribute to this asymptomatic persistence and evasion of antibiotic therapy. These hypotheses, as well as the mechanism leading to the re-emergence of the persistent strain after such a long period of asymptomatic carriage, remain to be explored.

## Concluding Remarks and Future Perspective

**Figure 2:**
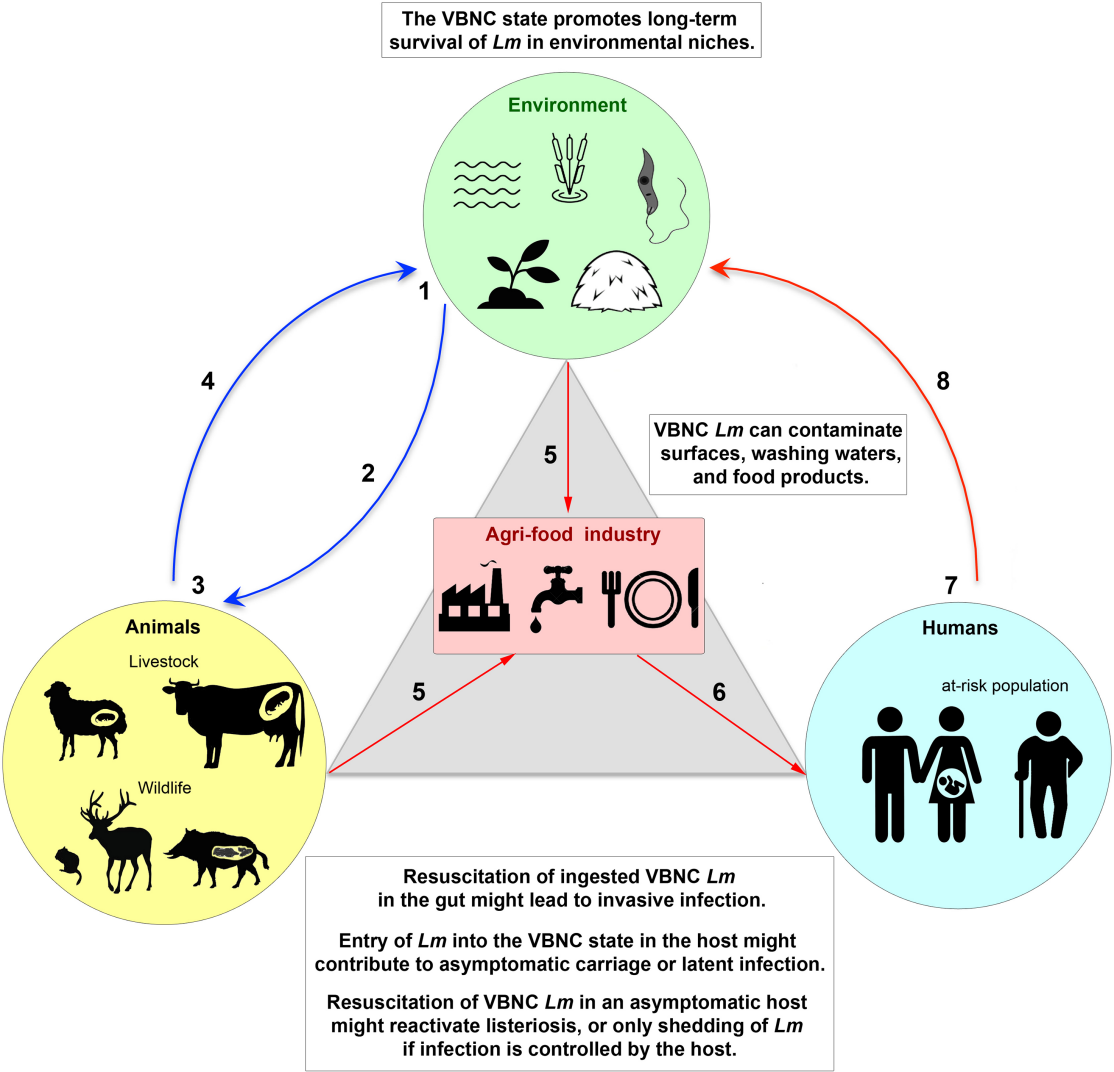
The VBNC state of *L. monocytogenes* in the One Health *continuum*. (1) *Lm* is present in a wide range of environmental ecosystems (e.g., sea, rivers, lakes, soil, plants, fodder, and possibly within unicellular eukaryotes), either in a vegetative culturable state (VC) or in a viable but non-culturable (VBNC) dormant state that allows long-term survival under hostile conditions. (2) VC and VBNC bacteria are ingested by animals, in particular farmed and wild mammals, *via* food or water. (3) VBNC forms could be reactivated into VC forms in the gut by a resuscitation process. Active VC forms can lead to invasive infection or enter a steady state with the host, resulting in long-term asymptomatic carriage. Entry of bacteria into the VBNC state could contribute to this asymptomatic carriage, by promoting silent colonization of the gastrointestinal tract (gut-liver axis) or the female reproductive system. Resuscitation of dormant VBNC *Lm* in an asymptomatic host would reactivate a latent infection, leading to listeriosis or, if the infection is controlled by the host, to shedding of *Lm* into the environment. (4) Excretion of *Lm* from animals, in feces, abortive products or cadavers, releases the pathogen into the environment, initiating a new cycle. (5) During processing of animal or plant products, *Lm* can contaminate food industry production lines. Antimicrobial treatments and/or biofilm formation favor the appearance of VBNC bacteria and their persistence on surfaces, process wash waters, or in food matrices. VBNC *Lm* can thus contaminate raw or ready-to-eat foods. (6) Humans become infected by eating foods contaminated with VC or VBNC *Lm*. (7) As in animals, resuscitation of VBNC *Lm* in the gut might cause invasive listeriosis, mainly in at-risk populations (pregnant women, the elderly or immunocompromised persons). The VC *Lm* forms might also switch to the VBNC state in tissues, resulting in asymptomatic colonization of healthy carriers or a latent infection phase in at-risk individuals. (8) Human shedding of *Lm* releases the pathogen into the environment.

The spread of human infectious diseases emerging from animal reservoirs, such as avian influenza caused by the H5N1 virus and Covid-19 caused by the SARS-CoV-2 virus, have brought to the forefront the fact that zoonotic diseases represent a major public health risk. It has also become clear that the asymptomatic carriage plays a key role in the transmission of these dangerous pathogens. Because of its ubiquity in the environment, animals, and humans, which are the three pillars of the One Health *continuum*, as well as in the agri-food industry that connects them (**Figure 2**), *Lm* represents an exceptional bacterial model to address these issues, including the mechanism of circulation of this pathogen in a chain of contamination involving different ecosystems. In addition to vegetative culturable *Lm* cells, dormant VBNC *Lm* need further investigation, as entry and exit from the VBNC state is likely one of the key mechanisms for *Lm* adaptation to stress in these different ecological niches, as are spores for spore-forming bacterial species. Since VBNC *Lm* forms are ubiquitous in the One Health *continuum* (**Figure 2**), their non-detection by conventional analytical methods poses a threat to public health and food safety. In the future, the implementation of a One Health approach to assess the impact of VBNC *Lm* in infection should be based on: (*i*) a more systematic sampling process in interconnected systems, which are animal and plant production systems (farms and their immediate environment), food production systems (food processing/packaging sites and food stores), and medical and hospital systems (samples from symptomatic listeriosis patients and at-risk asymptomatic individuals), (*ii*) the use of diagnostic methods without growth-based enrichment steps, to detect the presence of unculturable *Lm*, and (*iii*) whole-genome sequencing of isolated strains and comparative genomics, in order to determine which strains are most likely to enter the VBNC state, their ability to circulate, and in which ecosystem.

In the context of the food industry, safety standards concerning *Lm* contamination are all based on the growth capacity of this pathogen *via* the determination of CFU, both in the United States, with a zero-tolerance policy, and in Europe, with slightly less strict criteria for certain categories of RTE foods considered low risk (EC Regulation 2073/2005). These standards do not take into account the potential risk related to the presence of VBNC forms. As eradication of VBNC bacteria is difficult, their presence should be taken into account in risk assessment modeling and health regulation issues. In the context of VBNC bacteria in the host, many questions arise. For example, what are the physiological similarities and differences between bacteria in the VBNC state in an environmental niche and in a host? Are there strain-specific variations, with hypervirulent clones also more prone to silently persist as VBNC forms in the host, compared to hypovirulent clones? Do VBNC *Lm* survive asymptomatically in a host while being silently excreted through fecal or uterine discharges until the host dies, or can they reactivate in the same individual when the immune system is weakened, which would be consistent with latent listeriosis (**Figure 2**)? What would be the mechanisms of immune tolerance by the host of these VBNC bacterial parasites? What are the reservoir tissues (*e.g.*, intestine, liver, gallbladder, endometrium)? What are the reservoir hosts (including protozoa)? Answering these questions will require clinically relevant animal models and overcoming the problem of obtaining tissue samples from asymptomatic hosts. Additionally, in medicine, as in the agri-food industry, several challenges remain to be addressed, first of all, the development of sensitive, easy-to-use and inexpensive techniques to facilitate large-scale detection and tracing of VBNC *Lm*. Detection techniques based on PCR, DNA sequencing, or mass spectrometry, are improving and their development is crucial, as is the development of techniques demonstrating the viability of these non-culturable microorganisms. Second, it is necessary to identify the factors affecting the recovery and regrowth of VBNC cells. Finally, the pathogenic potential of VBNC forms must be properly assessed.

## Author Contributions

AL, HB: Writing–original draft preparation. AL, EM, HB: Writing–review and editing. AL, EM: Table preparation. HB: Figure preparation. EM, HB: Funding acquisition. HB: Supervision and Conceptualization. All authors contributed to the article and approved the submitted version.

## Funding

This work was funded by grants from ANR PERMALI (N°ANR-20-CE35-0001-01) and iXcore Foundation (2015) to HB, and INRAE-MICA division (AAP 2019-2020) to EM.

## Conflict of Interest

The authors declare that the research was conducted in the absence of any commercial or financial relationships that could be construed as a potential conflict of interest.

## Publisher’s Note

All claims expressed in this article are solely those of the authors and do not necessarily represent those of their affiliated organizations, or those of the publisher, the editors and the reviewers. Any product that may be evaluated in this article, or claim that may be made by its manufacturer, is not guaranteed or endorsed by the publisher.
